# Characteristic mutations induced in the small intestine of *Msh2*-knockout *gpt* delta mice

**DOI:** 10.1186/s41021-021-00196-0

**Published:** 2021-07-05

**Authors:** Yasunobu Aoki, Mizuki Ohno, Michiyo Matsumoto, Michi Matsumoto, Kenichi Masumura, Takehiko Nohmi, Teruhisa Tsuzuki

**Affiliations:** 1 Health and Environmental Risk Division, National Institute for Environmental Studies, 16-2 Onogawa, Tsukuba, Ibaraki, 305-8506 Japan; 2 Kyushu University, Faculty of Medical Sciences, Maidashi, Higashi-ku, Fukuoka, 812-8582 Japan; 3 Division of Genetics and Mutagenesis, National Institute of Health Sciences, Tonomachi, Kawasaki-ku, Kawasaki, Kanagawa 210-9501 Japan

**Keywords:** Mismatch repair, Oxidative stress, Potassium bromate, Single-base deletion, Transgenic rodent assay, Tumorigenesis

## Abstract

**Background:**

Base pair mismatches in genomic DNA can result in mutagenesis, and consequently in tumorigenesis. To investigate how mismatch repair deficiency increases mutagenicity under oxidative stress, we examined the type and frequency of mutations arising in the mucosa of the small intestine of mice carrying a reporter gene encoding guanine phosphoribosyltransferase (*gpt*) and in which the *Msh2* gene, which encodes a component of the mismatch repair system, was either intact (*Msh2*+/+::*gpt*/0; *Msh2*-bearing) or homozygously knockout (KO) (*Msh2*−/−::*gpt*/0; *Msh2*-KO).

**Results:**

*Gpt* mutant frequency in the small intestine of *Msh2*-KO mice was about 10 times that in *Msh2*-bearing mice. Mutant frequency in the *Msh2*-KO mice was not further enhanced by administration of potassium bromate, an oxidative stress inducer, in the drinking water at a dose of 1.5 g/L for 28 days. Mutation analysis showed that the characteristic mutation in the small intestine of the *Msh2*-KO mice was G-to-A transition, irrespective of whether potassium bromate was administered. Furthermore, administration of potassium bromate induced mutations at specific sites in *gpt* in the *Msh2*-KO mice: G-to-A transition was frequently induced at two known sites of spontaneous mutation (nucleotides 110 and 115, CpG sites) and at nucleotides 92 and 113 (3′-side of 5′-Gp**G**-3′), and these sites were confirmed to be mutation hotspots in potassium bromate-administered *Msh2*-KO mice. Administration of potassium bromate also induced characteristic mutations, mainly single-base deletion and insertion of an adenine residue, in sequences of three to five adenine nucleotides (A-runs) in *Msh2*-KO mice, and elevated the overall proportion of single-base deletions plus insertions in *Msh2*-KO mice.

**Conclusions:**

Our previous study revealed that administration of potassium bromate enhanced tumorigenesis in the small intestine of *Msh2*-KO mice and induced G-to-A transition in the *Ctnnb1* gene. Based on our present and previous observations, we propose that oxidative stress under conditions of mismatch repair deficiency accelerates the induction of single-adenine deletions at specific sites in oncogenes, which enhances tumorigenesis in a synergistic manner with G-to-A transition in other oncogenes (e.g., *Ctnnb1*).

**Supplementary Information:**

The online version contains supplementary material available at 10.1186/s41021-021-00196-0.

## Introduction

Base pair mismatches in genomic DNA can result in mutagenesis, and consequently in tumorigenesis [1]. Oxidative stress is known to accelerate mutagenesis via the formation of oxidative DNA adducts (e.g., 8-oxo-deoxyguanosine (8-oxo-dG)), resulting in tumorigenesis, especially when the systems for repairing base pair mismatches are deficient [2]. Two DNA repair systems are involved in preventing the mutagenesis caused by the formation of 8-oxo-dG: the base excision repair system [3], which is initiated by 8-oxoguanine glycosylase or mutY adenine DNA glycosylase, and the mismatch repair system, which involves the proteins MutS homolog (MSH) 2, 3, and 6 [4–6]. Currently, our understanding of the mutational mechanisms crucial for tumorigenesis under oxidative stress, and of how the two DNA repair systems contribute to suppressing tumorigenesis, is incomplete.

In a study to address how the base excision repair system acts to suppress tumorigenesis, Isoda et al. [6] demonstrated that the administration of 2 g/L potassium bromate in drinking water for 16 weeks to increase oxidative stress induced the development of tumors in the small intestine of *Mutyh*-knockout (KO) mice. In these mice, the activity of the base excision repair system to remove adenine misincorporated opposite 8-oxo-dG is suppressed. As a result, G-to-T transversions, which are a landmark mutation of oxidative stress, were observed in tumor tissue, particularly in the 5′-TGAA-3′ sequence of the *Apc* gene, which encodes a component of the Wnt signaling pathway. Recently, our group showed that 90-day administration of 0.6 g/L potassium bromate in drinking water increased mutant frequency in the small intestine of *gpt* (guanine phosphoribosyltransferase) delta mice [7], in which the *gpt* gene of *Escherichia coli* carried on lambda EG10 phage DNA (80 copies per haploid) is integrated into the mouse genomic DNA as a target gene for detecting mutations in vivo [8, 9]. Specifically, we found that the frequency of G-to-T transversion was increased, particularly in a 5′-TGAA-3′ sequence in the *gpt* gene [7]. Together, these observations suggest that G-to-T transversion in the 5′-TGAA-3′ sequence of the *Apc* gene is an initiating event of tumorigenesis in the small intestine via disruption of the Wnt signaling pathway.

Piao et al. [5] have reported that tumorigenesis is also induced in the small intestine of *Msh2*-KO mice, in which mismatch repair activity is suppressed, and is dramatically enhanced by the administration of potassium bromate under the same conditions as those used in the experiment of Isoda et al. using *Mutyh*-KO mice [6]. Mutation analysis of the oncogenes in tumors from these mice revealed induction of G-to-A transitions in the *Ctnnb1* gene, which encodes β-catenin, a protein involved in the canonical Wnt signaling pathway. This suggests that G-to-A transition in *Ctnnb1* plays a role in tumorigenesis in the small intestine under MSH2 deficiency or oxidative stress. However, this finding was unexpected, because G-to-T transversion, not G-to-A transition, is currently recognized as the mutation most frequently induced under oxidative stress [2, 10, 11]. Thus, it remains to be clarified whether G-to-A transition or G-to-T transversion is the most frequent base substitution under MSH2 deficiency and/or oxidative stress.

Gene-KO *gpt* delta mice are a useful tool for examining the contribution of a single gene to the suppression of mutagenesis in vivo. For example, we previously established Nrf2 (a transcription factor regulating the gene expression of phase II drug-conjugating enzymes and antioxidant proteins)-deficient *gpt* delta mice, and demonstrated that intratracheal administration of benzo[*a*]pyrene, a potent environmental mutagen, significantly increased mutation frequency in the murine lung [12].

In the present study, we developed *Msh2*-KO mice carrying the *gpt* gene, and examined which mutations were frequently induced in the small intestine, the target organ of tumorigenesis, under conditions of mismatch repair system deficiency. Initially, we thought that it would be impossible to produce *Msh2*-KO *gpt* delta mice because the *Msh2* gene and the site for the integration of the *gpt* gene are both located on the same chromosome (i.e., chromosome 17). However, mating of heterozygous *Msh2*-KO mice with *gpt* delta mice produced *Msh2*-KO mice carrying the *gpt* gene, probably as a result of homologous recombination. We used these mice to examine the *gpt* mutations that arose in the mucosa of the small intestine. Mutation analysis showed that the characteristic mutation was G-to-A transition, irrespective of whether potassium bromate was administered. We also found that administration of potassium bromate to *Msh2*-KO mice resulted in characteristic mutations, mainly single-base deletions and insertions of adenine, in sequences of three to five adenine nucleotides.

## Materials and methods

### Production of *Msh2*-KO and *Msh2*-bearing *gpt* delta mice

C57BL/6 J *Msh2*-KO mice were established as described previously [4] and *gpt* delta mice (C57BL/6 J background) [8] were obtained from Japan SLC (Shizuoka, Japan). Male heterozygous MSH2-deficient mice (*Msh2*+/−) were crossed with female *gpt* delta transgenic mice (*Msh*2+/+::gpt/gpt), and the resultant F1 heterozygous MSH2-deficient mice carrying the *gpt* gene (*Msh*2+/−::gpt/0) were crossed again with heterozygous MSH2-deficient mice (*Msh*2+/−). From the resultant offspring of backcrossing, mice with homozygous *Msh2* knockout but carrying the *gpt* gene (*Msh2*−/−::gpt/0; *Msh2*-KO mice) and mice carrying the *Msh2* wild-type allele and the *gpt* gene (*Msh2*+/+::gpt/0; *Msh2*-bearing mice) were used in the present study.

Genotyping for *Msh2* was accomplished by polymerase chain reaction (PCR) amplification of genomic DNA isolated from tissue sampled from the tails of the mice. PCR amplification was conducted as follows:

1. A 220-base-pair (bp) amplicon was produced with Primers 1 and 2 detecting the *Msh2* wild-type allele, and a 450-bp amplicon was produced with Primers 3 and 4 detecting the *Msh2*-KO allele [4]. These amplicons were visualized by UV transilluminator (Printgraph TP-20MP, ATTO CORPORATION, Tokyo, Japan) after separating on an agarose gel (1%) and staining with ethidium bromide.

Primer 1: 5′-GTAATTATGCGTTTCAGGTCAG-3′ (forward).

Primer 2: 5′-GCGCTGTGACATGTAGATTATT-3′ (reverse) targeting *Msh2* gene exon 14.

Primer 3: 5′-GATTGCACGCAGGTTCTCCG-3′ (forward for *neo*^*r*^ gene).

Primer 4: 5′-GCTCTTCGTCCAGATCATCC-3′ (reverse for *neo*^*r*^ gene) targeting the polII–neo–poly(A) cassette.

2) A 2000-bp amplicon and a 1000-bp amplicon was produced from *Msh2* wild-type allele (Primers 5 and 6) and *Msh2*-KO allele (Primers 6 and 7), respectively [4]. The amplicons were visualized by UV transilluminator (Optima Shot OS-300, Wako, Osaka, Japan) after separating on the agarose gel as described above.

Primer 5: 5′-CCTGTGAGTCGGCAGAAG-3′.

Primer 6: 5′-CGGGAAGTTAGCGAGCTC-3′.

Primer 7: 5′-TGCAATCCATCTTGTTCAATG-3′.

Genotyping for *gpt* was accomplished by PCR amplification of the same genomic DNA as used for the *Msh2* genotyping but using the following PCR primers as previously reported [13]:

5′-GTTGTACTTCCAACCATGCCAAAG-3′ (sense for both genotypes).

5′-CAGAAATCATTCCAGGTCCTTGC-3′ (antisense for wild-type mice).

5′-CCCAGGTAATGAATAATTGCCTGTTTG-3′ (antisense for *gpt*).

As results of PCR genotyping, seven *Msh2*-KO and nine *Msh2*-bearing *gpt* delta mice were selected. Representative *Msh2* genotyping results are shown in Additional Fig. S1.

To confirm knockout of the *Msh2* gene, whole-genome sequencing was conducted for one of the *Msh2*-KO mice (animal #1 in the vehicle control group, see Additional Table S1). The genomic DNA extracted from the mucosa of the small intestine by a method described below in a section of ‘Extraction of genomic DNA’ was subjected to whole-genome sequencing. Sequencing and data analysis were performed on our behalf by Macrogen Japan Corp. (Kyoto, Japan). A DNA library for the whole genome sequence was constructed in accordance with the TruSeq DNA PCR-Free Library Preparation Guide (Illumina, Inc. San Diego, CA, USA), and sequencing was accomplished on a NovaSeq 6000 system (Illumina, Inc.) using 150-bp paired ends. Sequence reads were mapped to the mouse reference sequence (C57BL/6 J, NCBI Build 38, mm10) by using Isaac aligner. Single nucleotide variants and short indels were called by using the Isaac variant caller. The status of the *Msh2*-KO allele was confirmed by using Integrative Genomics Viewer and the read depth at the *Msh2* genomic region was validated. Additional Fig. S2 shows the targeted region of the *Msh2* gene on chromosome 17 that was knocked out in this mouse.

To identify the lambda EG10 insertion site, sequenced reads were re-aligned against the reference sequence (mm10 sequence + lambda EG10 sequence) by using Burrows–Wheeler Aligner. Chimeric reads and chimeric paired reads were mapped to mm10 sequence + lambda EG10 sequence, and the position of each read was visualized in Integrative Genomics Viewer. Multiple chimeric reads were mapped on chromosome 17qB2, and the integration sites were chr17:40741861 and chr17:40741930 (Additional Fig. S3), which were consistent with the integration sites reported for the mm9 reference genome [13]. Thus, the whole-genome sequencing data confirmed the PCR genotyping results.

### Administration of potassium bromate

Potassium bromate (Sigma-Aldrich, St. Louis, MO, USA) was orally administered to *Msh2*-bearing mice and *Msh2*-KO mice (6 weeks old, *n* = 3–5, male or female) for 28 days at a dose of 0 or 1.5 g/L in the distilled water, in accordance with OECD Test No. 488: Transgenic Rodent Somatic and Germ Cell Gene Mutation Assays, with minor modifications [14]. At the end of treatment, water without potassium bromate was provided ad libitum for 3 days, and then the animals were necropsied.

All animal experiments were performed according to protocols approved by the Institutional Animal Care and Use Committee at the National Institute for Environmental Studies, Japan.

### Collection of tissue

One-third of the small intestine on the stomach side (length approx. 10 cm) was excised from each mouse, flushed with Dulbecco’s phosphate-buffered saline (PBS; Nissui, Tokyo, Japan), and cut open as previously reported [7]. After rinsing gently with PBS to remove any remaining contents and mucus, the mucosa was separated from the intestinal wall by gentle scraping. The collected mucosa was frozen in liquid nitrogen and kept at − 80 °C until use.

### Extraction of genomic DNA

The *gpt* mutation assay was performed as previously reported [7, 8]. Briefly, genomic DNA was extracted from the mucosa of the small intestine by using a RecoverEase DNA Isolation Kit (Agilent Technologies, Santa Clara, CA, USA).

### *Gpt* mutation assay and sequencing of mutated *gpt*

From the extracted genomic DNA, lambda EG10 phages were recovered with Transpack Packaging Extract (Agilent Technologies). Next, *E. coli* YG6020 cells were infected with the recovered phages, plated on M9 salt plates containing chloramphenicol (Nacalai Tesque, Kyoto, Japan) and 6-thioguanine (6-TG, Nacalai Tesque) or chloramphenicol alone, and then incubated for 72 to 90 h at 37 °C. To confirm the phenotype, the 6-TG–resistant mutant colonies were collected and again streaked on plates containing chloramphenicol and 6-TG and incubated for 72 h at 37 °C [7, 8].

To confirm the *gpt* mutant, 6-TG–resistant *E. coli* were cultured overnight at 37 °C in Luria–Bertani broth containing 25 μg/mL chloramphenicol, harvested by centrifugation (7000 rpm, 10 min), and then stored at − 80 °C. A 739-bp DNA fragment containing *gpt* was amplified by PCR (primers 5′-TACCACTTTATCCCGCGTCAGG-3′ and 5′-ACAGGGTTTCGCTCAGGTTTGC-3′) and sequenced to identify the mutation in the *gpt* gene, as described previously [8, 15].

The *gpt* mutant frequency in each mouse was calculated by dividing the number of *gpt* mutant colonies growing on the agar plates containing chloramphenicol and 6-TG by the number of colonies growing on the agar plates containing chloramphenicol alone (titer). The frequency of each type of mutation was calculated by dividing the number of each mutation in each group by titer.

In this study, to examine whether the increase in mutant frequency was a result of clonal expansion in the mucosa of the small intestine, we estimated clonally corrected mutation frequency termed as ‘independent mutation frequency’, which was calculated by dividing the number of independent mutations occurred on *gpt* by titer.

### Statistical analysis

All data are expressed as means with standard deviation (SD). Differences were examined by using Tukey’s test for comparison among different treatment groups otherwise stated in the text; *P* < 0.05 was considered significant.

## Results

### Mutation assay

To examine how MSH2 deficiency affects mutagenicity induced by oxidative stress, we induced oxidative stress in *Msh2*-KO mice by providing the mice with potassium bromate in their drinking water and then examined the mutant frequency in the mucosa of the small intestine.

Potassium bromate was administered at 1.5 g/L in the drinking water for 28 days to five *Msh2*-bearing mice and four *Msh2*-KO mice, and distilled water without potassium bromate was provided to four *Msh2*-bearing mice and three *Msh2*-KO mice as vehicle controls (Additional Table S1 shows the sex of each animal). To examine the tumorigenicity of potassium bromate, 2 g/L of the agent in the drinking water for 16 weeks was initially used [5, 6], but the mice occasionally showed enervation. Therefore, we decided to use the lower concentration of potassium bromate that we found to still effectively induce tumors in the small intestine over 16 weeks (i.e., 1.5 g/L; Ohno, M., unpublished data). In the present study, potassium bromate at 1.5 g/L had no overt effect on body weight, and macroscopic observation revealed no anomalies in the small intestine in any of the treatment groups, which are observations consistent with those we reported previously [5].

**Fig. 1:**
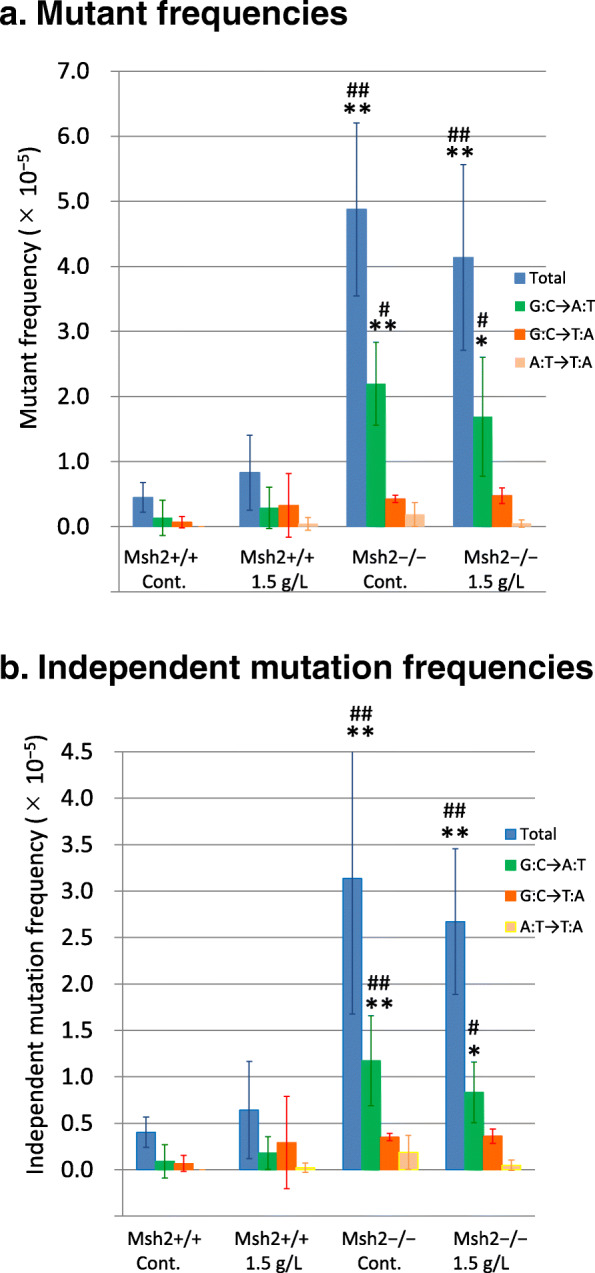
(**a**) Mutant frequencies and (**b**) independent mutation frequencies in the small intestine of *Msh2*-bearing (Msh2+/+) and *Msh2*-KO (Msh2−/−) mice carrying *gpt*. Potassium bromate was orally administered to the mice via the drinking water (1.5 g/L) for 28 days, or the vehicle control (Cont.). Independent mutation frequency is defined as frequency of independent mutants. Blue bars, total frequency; green bars, frequency of G-to-A transition; red bars, frequency of G-to-T transversion; yellow bars, frequency of A-to-T transversion. Data are presented as mean ± SD. * *P* < 0.05, ** *P* < 0.01, significantly different compared with Cont. of *Msh2*-bearing *gpt* delta mice. ^#^ *P* < 0.05, ^##^ *P* < 0.01, significantly different compared with *Msh2*-bearing *gpt* delta mice administered potassium bromate.

In *Msh2*-bearing mice, administration of 1.5 g/L potassium bromate increased the average total mutant frequency compared with vehicle control (0.83 ± 0.58 vs. 0.45 ± 0.23 × 10^− 5^), but this increase was not statistically significant (Fig. 1a and Additional Table S1). This finding was not consistent with our previous observation of a significant increase in mutant frequency after administration of potassium bromate at the higher dose of 2 g/L in the drinking water for 28 days [16].

Among the vehicle control mice, the average total mutant frequency was significantly higher in *Msh2*-KO mice than in *Msh2*-bearing mice (4.87 ± 1.33 vs. 0.45 ± 0.23 × 10^− 5^; *P* < 0.01). However, administration of potassium bromate did not cause any further significant increase in the total mutant frequency in these mice. Among the mice administered potassium bromate, the total mutant frequency was significantly higher in *Msh2*-KO mice than in *Msh2*-bearing mice (4.14 ± 1.43 vs. 0.83 ± 0.58 × 10^− 5^; *P* < 0.01).

To examine whether the increase in total mutant frequency in *Msh2*-KO mice was a result of clonal expansion in the mucosa of the small intestine, we estimated clonally corrected mutation frequency (i.e., the frequency at which an independent mutation occurred) in each group (Fig. 1b). Again, the total independent mutation frequency was significantly higher in the potassium bromate-administered and vehicle control *Msh2*-KO mice compared with the treatment-matched *Msh2*-bearing mice (2.67 ± 0.79 vs. 0.64 ± 0.53 × 10^− 5^, *P* < 0.01 and 3.14 ± 1.46 vs. 0.40 ± 0.16 × 10^− 5^, *P* < 0.01; respectively), and the total independent mutation frequency was not significantly changed by administration of potassium bromate in *Msh2*-KO mice compared with vehicle controls. These results indicate that the increase in total mutant frequency in the *Msh2*-KO mice was not a result of clonal expansion.

### Analysis of mutations

Next, we examined the base substitutions and other mutations induced in the mucosa of the small intestine by sequencing mutated *gpt* DNA and estimating the mutant frequencies. In *Msh2*-bearing mice, the frequencies of G-to-A transition and G-to-T transversion were comparable in both the vehicle control group (0.14 ± 0.27 × 10^− 5^ and 0.07 ± 0.09 × 10^− 5^, respectively) and the potassium bromate-administered group (0.29 ± 0.32 × 10^− 5^ and 0.33 ± 0.49 × 10^− 5^, respectively) (Fig. 1a). There was no significant increase in the frequencies of both base substitutions in the bromate-administered group compared with the vehicle control group. As oral administration of potassium bromate has already been shown to cause an increase in the frequency of G-to-T transversion in *gpt* delta mice under two different experimental conditions (2 g/L for 28 days [16] and 0.6 g/L for 90 days [7]), we suspect that the lack of a significant increase in the frequency of G-to-T transversion was a result of the dose of potassium bromate in the present study being insufficient to induce this base substitution. In contrast, the frequency of G-to-A transition in vehicle control *Msh2*-KO mice was significantly higher than that in vehicle control *Msh2*-bearing mice (2.20 ± 0.64 vs. 0.14 ± 0.27 × 10^− 5^; *P* < 0.01), and an increase in the mutant frequency of G-to-T transversion was observed in vehicle control *Msh2*-KO mice (0.43 ± 0.05 × 10^− 5^ vs. vehicle control *Msh2*-bearing mice; 0.07 ± 0.09 × 10^− 5^), but this increase was not statistically significant. In addition, administration of potassium bromate to the *Msh2*-KO mice did not cause any further increase in the frequencies of G-to-A transition (1.69 ± 0.91 × 10^− 5^) and G-to-T transversion (0.48 ± 0.12 × 10^− 5^).

In all of the treatment groups, G-to-A transition was the most abundant base substitution (Table 1). Even though the proportion of G-to-T transversion was increased by administration of potassium bromate in both *Msh2*-bearing mice (20% [2/10] to 29% [8/28]) and *Msh2*-KO mice (9% [10/107] to 13% [19/149]), G-to-A transition remained the most abundant base substitution. A-to-G transition was a rare base substitution in *Msh2*-bearing mice, but was more frequent in *Msh2*-KO mice; the proportion of A-to-G transition was increased by administration of potassium bromate in both *Msh2*-bearing mice (0% [0/10] to 7% [2/28]) and *Msh2*-KO mice (12% [13/107] to 17% [25/149]). This increase in A-to-G transition was consistent with our previous observations in the spleen of MSH2-deficient mice [4]. In addition, we previously reported that the major mutation in *Ctnnb1* in tumors from potassium bromate-administered *Msh2*-KO mice was G-to-A transition and that A-to-G transition and G-to-T transversion mutations were much less common [5].

**Table 1:** Intestinal *gpt* mutations in *Msh2*-bearing *(Ms2(+/+))* or *Msh2*-KO *(Msh2(-/-))* mice carrying *gpt* to which potassium bromate in the drinking water at a dose of 0 or 1.5 g/L was administered for 28 days

	*Msh2****(+/+)***				*Msh2****(−/−)***			
	0 g/L		1.5 g/L		0 g/L		1.5 g/L	
**Type of mutation in** ***gpt***	**Number**	**%**	**Number**	**%**	**Number**	**%**	**Number**	**%**
**Base substitution**								
**Transition**								
**G:C → A:T**	**3**	**30**	**10**	**36**	**51**	**48**	**60**	**40**
**(CpG site)**	**(1)**		**(8)**		**(20)**		**(35)**	
**A:T → G:C**	**0**	**0**	**2**	**7**	**13**	**12**	**25**	**17**
**Transversion**								
**G:C → T:A**	**2**	**20**	**8**	**29**	**10**	**9**	**19**	**13**
**G:C → C:G**	**0**	**0**	**1**	**4**	**0**	**0**	**1**	**1**
**A:T → T:A**	**0**	**0**	**2**	**7**	**4**	**4**	**2**	**1**
**A:T → C:G**	**1**	**10**	**0**	**0**	**6**	**6**	**2**	**1**
**Deletion**								
**1**	**2**	**20**	**2**	**7**	**17**	**16**	*26	**17**
**≧2**	**1**	**10**	**3**	**11**	**0**	**0**	**0**	**0**
**Insertion**	**1**	**10**	**0**	**0**	**6**	**6**	*12	**8**
**Other**	**0**	**0**	**0**	**0**	**0**	**0**	**2**	**1**
***Total***	***10***	***100***	***28***	***100***	***107***	***100***	***149***	***100***

Single-base deletion and insertion were more abundant in *Msh2*-KO mice administered potassium bromate (17% [26/149] and 8% [12/149], respectively) than in *Msh2*-bearing mice administered potassium bromate (7% [2/28] and 0% [0/28], respectively). The proportion of single-base deletion plus insertion in potassium bromate-administered *Msh2*-KO mice (26% [(26 + 12)/149]) was significantly higher than that in potassium bromate-administered *Msh2*-bearing mice (7% [(2 + 0)/28]) (*P* < 0.05, Fisher’s exact test).

### Identification of mutation hotspots

The advantage of using an in vivo mutagenesis assay system in transgenic rodents is that it can reveal not only the frequency of mutations but also the nucleotides that are most frequently mutated (mutation hotspots) and the nucleotide sequences near the points of mutation (mutational signatures). Here, we defined a “mutation hotspot” as the nucleotides for which a mutation was induced in three or more mice in a treatment group [7, 17–19].

**Fig. 2:**
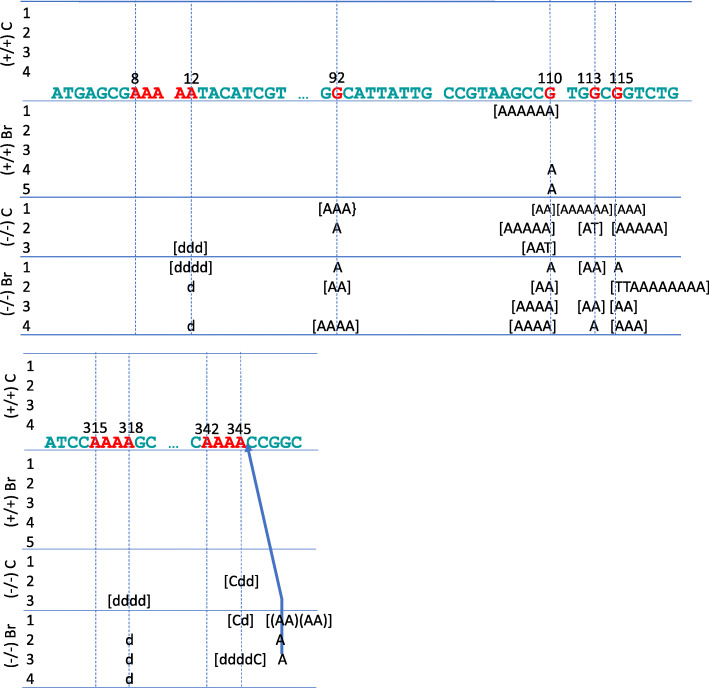
Mutation hotspots in the *gpt* gene in the small intestine of *Msh2-*bearing mice (+/+) carrying *gpt* and *Msh2*-KO mice (−/−) carrying *gpt*. Potassium bromate (1.5 g/L) (Br) in the drinking water or the vehicle control (C) was administered for 28 days. Numbers in the column next to the groups of animals are animal identification numbers shown in Additional Table S1. The letters A, G, T, and C indicate the bases substituted or inserted; and d and (AA) indicates deletion of base and insertion of two adenine residues, respectively. The number of letters in brackets are the number of clonal mutants. A blue arrow indicates the position of base insertion. Light blue letters are the parts of *gpt* sequence, and red letters in the *gpt* sequence, the dashed lines and the number above the sequence indicate the nucleotides of mutation hotspots, the positions of mutation hotspots and the nucleotide number, respectively.

Four G-to-A transition mutation hotspots were identified, at nucleotides 92, 110, 113, and 115. Nucleotide 110 was identified as a mutation hotspot not only in potassium bromate-administered and vehicle control *Msh2*-KO mice, but also in potassium bromate-administered *Msh2*-bearing mice (Fig. 2; details of the mutations on the *gpt* gene are shown in Additional Fig. S4). In contrast, although G-to-A transitions at nucleotides 92, 113, and 115 were frequently induced in potassium bromate-administered and vehicle control *Msh2*-KO mice, these nucleotides were identified as mutation hotspots only in potassium bromate-administered *Msh2*-KO mice, suggesting that induction of these three mutations under the MSH2-deficient condition was accelerated by oxidative stress. Although G-to-A transitions at nucleotides 110 and 115 are recognized as spontaneous mutations induced at CpG sites [20, 21], in the present study, the base substitution at nucleotide 115 was identified only in the potassium bromate-administered and vehicle control *Msh2*-KO mice. In addition, the G-to-A transitions at nucleotides 92 and 113 were both induced at the 3′-side of guanine on 5′-Gp**G**-3′, not on CpG. The reason why G-to-A transition was the most abundant base substitution under MSH deficiency remains unclear, but our observations suggest that deficiency of MSH2 accelerates spontaneous induction of G-to-A transition.

Sequences of three to five adenines, referred to as “A-runs”, were identified as sites at which mutations were significantly induced in potassium bromate-administered *Msh2*-KO mice compared with vehicle controls (Table 2; details of the mutations in the *gpt* gene are shown in Additional Fig. S4). The proportion of mutants in A-runs in potassium bromate-administered *Msh2*-KO mice (30/149) was significantly higher compared to that in vehicle control *Msh2*-KO mice (12/107) (*P* < 0.05, Fisher’s exact test). To exclude the possibility that the increase in mutant in the A-runs in potassium bromate-administered *Msh2*-KO mice was caused by clonal expansion, we analyzed the proportion of independent mutations in these A-runs; we found that this proportion was also significantly higher in potassium bromate-administered *Msh2*-KO mice (21/97) than in the vehicle control *Msh2*-KO mice (6/66) (*P* < 0.05, Fisher’s exact test). The major mutations induced in the A-runs were single-base deletions and one- or two-base insertions of adenine, and the remaining mutations were A-to-G (T-to-C) transitions and A-to-C transversions. Single-base deletions in a five- (nucleotides 8–12) and a four-nucleotide A-run (nucleotides 315–318), and insertions in a four-nucleotide A-run (nucleotides 342–345) were identified as the major hotspot-related mutations in potassium bromate-administered *Msh2*-KO mice (Fig. 2). These results suggest that oxidative stress under MSH2 deficiency induces deletions, insertions, and several base substitutions in A-run sequences.

**Table 2:** Summary of mutations induced in sequences of three to five adenine nucleotides (A-runs) in the *gpt* gene in the small intestine of *Msh2*-KO mice

Nucleotide position of A-run within the *gpt* gene	Original sequence	Mutated sequence	Number of mutants and independent mutations	
			Vehicle control	KBrO_3_ administered
7–12	AAAAA	AAAAd	3 (1)	6 (3)
88–90	AAA	AAd	1 (1)	2 (1)
173–175	TTT	TTC	0	2 (1)
179–181	TTT	TTC	0	1 (1)
		TTTT	0	1 (1)
214–216	AAA	AAAA	0	1 (1)
223–225	AAA	AAd	1 (1)	2 (2)
315–318	AAAA	AAAd	4 (1)	3 (3)
325–327	TTT	TTd	0	1 (1)
342–345	AAAA	AAAd	2 (1)	5 (2)
		AAAAAA	0	2 (1)
		AAAAA	0	2 (2)
		AAAC	1 (1)	2 (2)
Number of mutants in A-runs			12(6)	30 * (21 *)
Total number of mutants in *gpt*			107 (66)	149 (97)

* *P* < 0.05, significantly different compared with vehicle control by Fisher’s exact test.

Numbers in parentheses indicate the numbers of independent mutations.

“d” indicates base deletion.

## Discussion

Here, we demonstrated that in vivo mutagenesis in the mucosa of the murine small intestine was significantly increased under MSH2 deficiency. The administration of potassium bromate to induce oxidative stress did not result in a further increase in mutagenesis in the *Msh2*-KO mice compared with that in vehicle controls, but deletions and insertions in A-runs were identified as characteristic mutations induced by administration of potassium bromate.

Mutation analysis revealed that the frequency of G-to-A transitions was significantly increased in potassium bromate-administered and in vehicle control *Msh2*-KO mice compared with that in *Msh2*-bearing mice (Fig. 1a), and that this base substitution was the most frequent mutation in *Msh2*-KO mice (Table 1). Interestingly, in *Msh2*-KO mice, we found that the frequency of G-to-T transversion, a landmark mutation of oxidative stress, was lower than that of G-to-A transition and was not increased by administration of potassium bromate (Fig. 1a). Analysis of independent mutation frequency revealed that the independent mutation frequency of G-to-A transition and the total independent mutation frequency were significantly increased in potassium bromate-administered and in vehicle control *Msh2-*KO mice (Fig. 1b), indicating that the frequency of G-to-A transition was not a result of clonal expansion in the small intestine induced by MSH2 deficiency. This increase in the frequency of G-to-A transition in *Msh2*-KO mice is consistent with our previous observations that G-to-A transition frequently occurred in *Ctnnb1* in tumors in the small intestine of *Msh2*-KO mice [5], and that the most frequent base substitution in *CTNNB1* in human colorectal tumors was G-to-A transition [22]. The mechanism underlying the observed frequent induction of G-to-A transition in *Msh2*-KO mice remains unknown; however, because G-to-A transition was not elevated by administration of potassium bromate, the enhanced tumorigenesis in *Msh2*-KO mice by potassium bromate cannot be explained simply by increased induction of this base substitution [5].

Next, we examined the possibility that site-specific mutations are enhanced by potassium bromate under MSH2-deficiency. By using DNA sequencing to determine the common mutation sites in *gpt*, we found that nucleotides 92, 110, 113, and 115 were mutation hotspots for G-to-A transition in potassium bromate-administered *Msh2*-KO mice (Fig. 2). Nucleotides 110 and 115 are located in CpG sites, in which G-to-A transition is known to spontaneously occur [18, 23, 24], suggesting that oxidative stress contributes to spontaneous induction of G-to-A transition at these CpG sites. In contrast, because nucleotides 92 and 113 are both guanines on the 3′ side of a 5′-Gp**G-**3′ site, not a CpG site, the DNA sequences around nucleotides 92 and 113 may be susceptible to oxidative stress in *Msh2*-KO mice. In our previous study, G-to-A transitions were detected in 5′-Gp**G-**3′ sites in *Ctnnb1* in 12 of 27 tumors induced in the small intestine of potassium bromate-administered Msh2-KO mice [5].

Mutation analysis also revealed that the mutations, mainly single-adenine deletions and adenine insertions, were induced in A-runs at a significantly higher frequency in potassium bromate-administered *Msh2*-KO mice than in vehicle controls (Table 2; *P* < 0.05, Fisher’s exact test). Among these A-runs, those at nucleotides 8–12, 315–318, and 342–345 were mutation hotspots of single-adenine deletions or adenine insertions in potassium bromate-administered *Msh2*-KO mice (Fig. 2). This finding is consistent with our observation of single-base deletions or insertions in four- or five-nucleotide A-runs in the small intestine of potassium bromate-administered *Msh2*-KO *rpsL*-integrated mice [4] produced by mating *Msh2*-KO mice with transgenic mice carrying the *E. coli rpsL* gene for the detection of mutations induced in organs in vivo [4, 25]. Together, these findings suggest that A-runs could be susceptible to mutation, especially single-adenine deletion or adenine insertion, in the small intestine of *Msh2*-KO mice under oxidative stress. Because the MSH2/MSH3 heterodimer assists in DNA repair by recognizing small deletions and insertions [26], it is possible that the deletion or insertion of adenine in A-runs caused by potassium bromate-inducing the formation of oxidative adenine adducts or the incorporation of 8-oxo-guanine opposite to adenine [2] failed to be repaired under MSH2 deficiency. However, the mechanism underlying the induction of mutations in A-runs by potassium bromate in *Msh2*-KO mice remains to be determined.

As mentioned above, we previously found that G-to-A transition occurred in *Ctnnb1* in tumors in the small intestine of potassium bromate-administered *Msh2*-KO mice [5]. Our present data indicates that G-to-A transition was the most frequent base substitution in both vehicle control and potassium bromate-administered *Msh2*-KO mice, but also that the frequency of this base substitution was not elevated by administration of potassium bromate. Together, these findings suggest that while the induction of G-to-A transition in *Ctnnb1* may be essential for tumorigenesis under MSH2 deficiency, mutations other than G-to-A transition in multiple oncogenes may also be required for potassium bromate to enhance tumorigenesis in the small intestine of *Msh2*-KO mice. Indeed, Deihimi et al. have demonstrated that the *BRCA2*, *EGFR*, and *NTRK* are mutated in human mismatch repair–deficient colorectal cancers with *MSH2* or *MLH1* mutations [27].

Hegan et al. have shown by mutation analysis using *supFG1* or *CII* (a transgene for detection of in vivo mutations) integrated in genomic DNA that the major mutations induced in the tissues of mismatch repair gene (*Pms2*, *Mlh1*, *Msh2*, *Msh3*, or *Msh6*)-deficient mice were deletions and insertions [28]. Single-cell whole-genome sequencing revealed also that the frequency of small base-deletions or insertions was elevated in fibroblasts derived from *Msh2*-KO mice compared with wild-genotype cells [29]. These findings indicate that base deletions and insertions tend to be induced under MSH2 deficiency. In the present study, administration of potassium bromate significantly increased the frequency of certain single-base deletions plus insertions in the small intestine of *Msh2*-KO mice compared to that in *Msh2*-bearing mice (Table 1), and increased the frequency of single-base deletions or insertions of adenine in A-runs in *Msh2*-KO mice (Table 2). Regarding tumorigenesis in humans, Vargas-Parra et al. have reported that adenine deletions are frequently found in the *APC*, *AXIN2*, *BMPR1A*, *PTEN*, or *BUB1B* oncogenes in mismatch repair-deficient tumors [30]. These observations suggest that increased frequency of deletion of adenine at specific sites (e.g., A-runs) enhances tumorigenesis induced by potassium bromate in the small intestine of *Msh2*-KO mice.

## Conclusions

Here, we demonstrated that G-to-A transition is the most frequent base substitution in the small intestine of *Msh2*-KO mice, but that the frequency of this base substitution was not elevated by administration of potassium bromate. Previously, we reported that G-to-A transition occurred in the *Ctnnb1* gene in tumors induced in the small intestine of *Msh2*-KO mice administered potassium bromate [5]. These observations suggest that G-to-A transition is a characteristic mutation under MSH2 deficiency, and that induction of this base substitution in *Ctnnb1* is a key event in tumorigenesis [31] in the small intestine of *Msh2*-KO mice, but the induction of mutations other than G-to-A transition was expected to enhance tumorigenesis by administration of potassium bromate because the administration of this agent did not cause any further increase in the frequencies of G-to-A transition. Our analysis of mutations in the *gpt* gene showed that single-base deletions and insertions of adenine were frequently induced in three- to five-nucleotide A-runs in potassium bromate-administered *Msh2*-KO. Indeed, the deletion of adenine has already been identified as a major mutation in the oncogenes in human mismatch repair-deficient tumors [30].

Taking our present and previous observations together, we propose that oxidative stress under conditions of mismatch repair-deficiency accelerates the induction of single-adenine deletions at specific sites in oncogenes, which enhances tumorigenesis in a synergistic manner with G-to-A transition in other oncogenes (e.g., *Ctnnb1*). Further studies are required to identify the events underlying the induction of mutagenesis and tumorigenesis by oxidative stress in the human intestine/colon.

## Supplementary Information

**Additional file 1: Figure S1**. Representative *Msh2* genotyping results. The genotype of each mouse was determined by polymerase chain reaction (PCR) using (a) the primer 1–4 and (b) the primer 5–7 described in Materials and Methods. WT and KO indicate the PCR product of the *Msh2* wild-type allele and *Msh2* KO allele, respectively, and numbers indicated in parenthesis are the size (bp, base pair) of amplicons. ‘+/+’, ‘+/−’, and ‘−/−’ indicate the *Msh2* genotype of each animal, as determined by PCR. Dose, A dose of potassium bromate (g/L) to which each mouse was administered. ID, ID of the animal in the treatment groups, as shown in Additional **Table S****1**.

**Additional file 2: Figure S2**. Screenshot of the *Msh2* genomic region of an *Msh2*-KO mouse (animal #1 in the vehicle control group; see also Additional Table S1) as shown in Integrative Genomics Viewer. There is no mapped sequence read for *Msh2* exon 13 to exon 14 corresponding to the targeted region that was described in the original paper reporting the establishment of *Msh2*-KO mice [4].

**Additional file 3: Figure S3**. Screenshot of the 17qB2 region of an *Msh2*-KO mouse (animal #1 in the vehicle control group; see also Additional Table S1) as shown in Integrative Genomics Viewer. All sequence reads were mapped against a reference sequence (mm10 + lambda EG10). Only chimeric reads (Lambda EG10: genomic or genomic: Lambda EG10) are shown in this figure. Junction 1 and 2 indicate presumed integration sites of the *gpt* gene. A junction is a site boundary between the mouse genome sequence and the Lambda EG10 sequence within a chimeric read. The two junctions mapped on the mm10 reference sequence were consistent with two previously reported junctions reported using the mm9 reference sequence [13].

**Additional file 4: Figure S4**. Positions of mutations in the *gpt* gene in the small intestine of *Msh2-*bearing mice (M(+/+)) carrying *gpt* and *Msh2*-KO mice (M(−/−)) carrying *gpt*. Potassium bromate (1.5 g/L) in the drinking water or the vehicle control (Control) was administered for 28 days. Numbers in the column next to the doses are animal identification numbers shown in Additional Table S1. Letters A, G, T, and C indicate base substitutions, and the number of letters in brackets are the number of clonal mutants. Arrows indicate the positions of insertion of base(s). d and (AA) indicates deletion of base and insertion of two adenine residues, respectively. Red letters in the *gpt* sequence indicate nucleotides whose mutations were induced.

**Additional file 5: Table S1**. Mutant frequencies in the small intestine of *Msh2*-bearing or -KO mice carrying *gpt* to which vehicle or potassium bromate was administered via the drinking water.

## Data Availability

All data generated or analyzed during this study are included in this published article and its supplementary information files.

## Abbreviations

gpt: Guanine phosphoribosyltransferase
KO: Knockout
MSH: MutS homolog
8-oxo-dG: 8-oxo-deoxyguanosine
PBS: Dulbecco’s phosphate-buffered saline
PCR: Polymerase chain reaction
6-TG: 6-thioguanine
