# Supplementary figures and images for: Characteristic mutations induced in the small intestine of Msh2-knockout gpt delta mice
Source: Genes Environ. 2021 Jul 5;43:27. doi: 10.1186/s41021-021-00196-0 (PMC8256579; doi:10.1186/s41021-021-00196-0)

Fig. S1

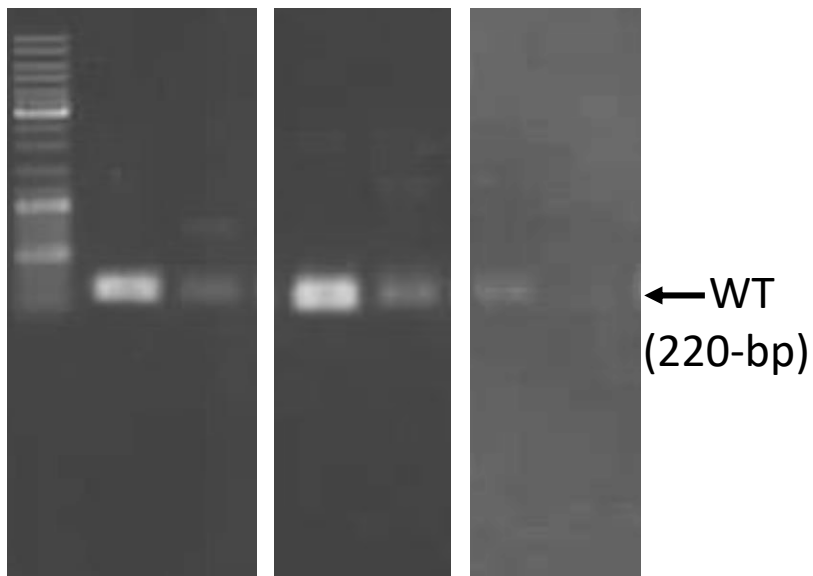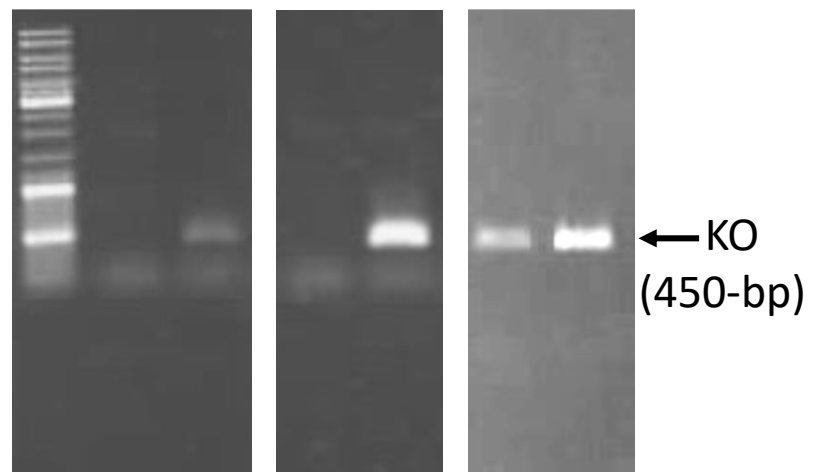

|          |     |     |     |     |     |     |
|----------|-----|-----|-----|-----|-----|-----|
| Genotype | +/+ | +/- | +/+ | +/- | +/- | -/- |
| Dose     | 0   |     | 1.5 |     |     | 1.5 |
| ID       | 1   |     | 2   |     |     | 3   |

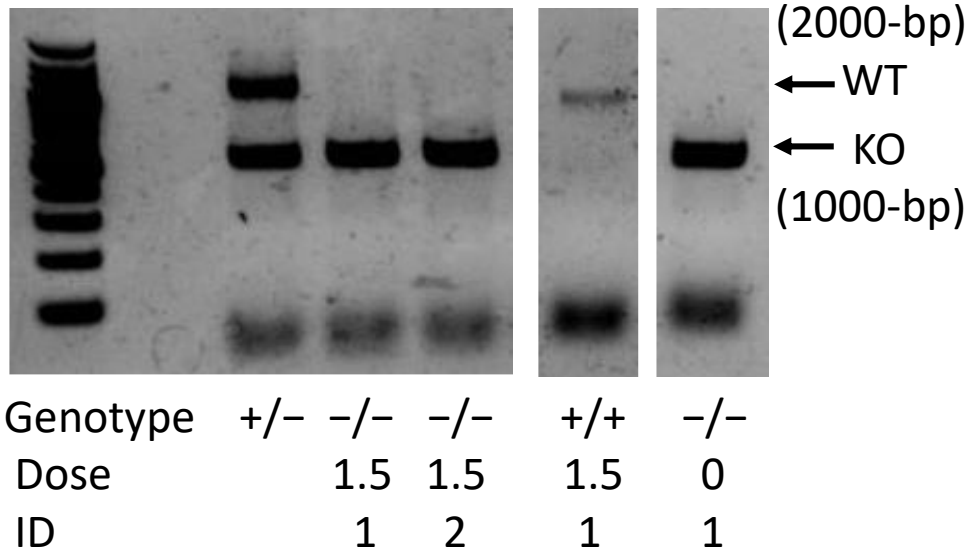

|          |     |     |     |     |     |
|----------|-----|-----|-----|-----|-----|
| Genotype | +/- | -/- | -/- | +/+ | -/- |
| Dose     |     | 1.5 | 1.5 | 1.5 | 0   |
| ID       |     | 1   | 2   | 1   | 1   |

Supplement: Supplementary file 1 — Additional file 1: Figure S1. Representative Msh2 genotyping results. The genotype of each mouse was determined by polymerase chain reaction (PCR) using (a) the primer 1–4 and (b) the primer 5–7 described in Materials and Methods. WT and KO indicate the PCR product of the Msh2 wild-type allele and Msh2 KO allele, respectively, and numbers indicated in parenthesis are the size (bp, base pair) of amplicons. ‘+/+’, ‘+/−’, and ‘−/−’ indicate the Msh2 genotype of each animal, as determined by PCR. Dose, A dose of potassium bromate (g/L) to which each mouse was administered. ID, ID of the animal in the treatment groups, as shown in Additional Table S1. [file 41021_2021_196_MOESM1_ESM.pdf]

## IGV region view: 17qE4

targeted region

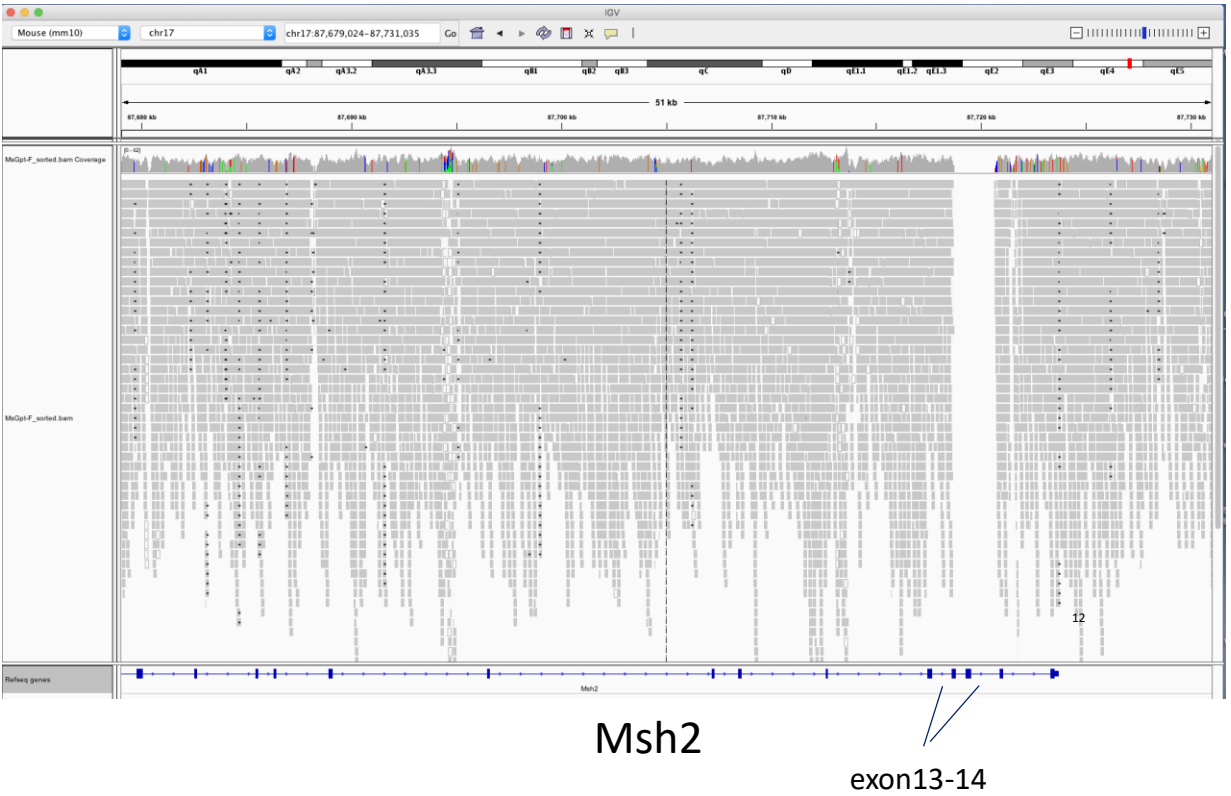

Fig. S2.

Supplement: Supplementary file 2 — Additional file 2: Figure S2. Screenshot of the Msh2 genomic region of an Msh2-KO mouse (animal #1 in the vehicle control group; see also Additional Table S1) as shown in Integrative Genomics Viewer. There is no mapped sequence read for Msh2 exon 13 to exon 14 corresponding to the targeted region that was described in the original paper reporting the establishment of Msh2-KO mice [4]. [file 41021_2021_196_MOESM2_ESM.pdf]

IGV region view: 17qB2

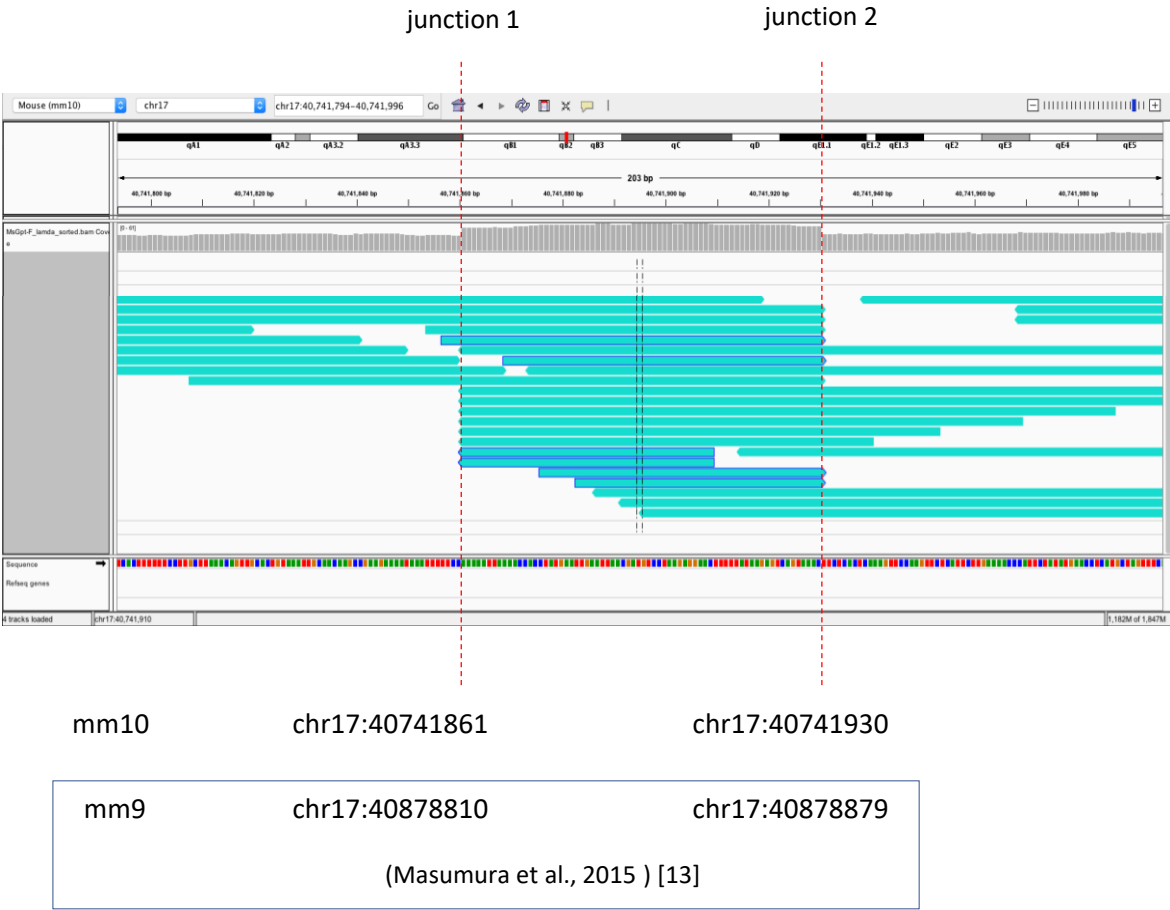

Fig. S3.

Supplement: Supplementary file 3 — Additional file 3: Figure S3. Screenshot of the 17qB2 region of an Msh2-KO mouse (animal #1 in the vehicle control group; see also Additional Table S1) as shown in Integrative Genomics Viewer. All sequence reads were mapped against a reference sequence (mm10 + lambda EG10). Only chimeric reads (Lambda EG10: genomic or genomic: Lambda EG10) are shown in this figure. Junction 1 and 2 indicate presumed integration sites of the gpt gene. A junction is a site boundary between the mouse genome sequence and the Lambda EG10 sequence within a chimeric read. The two junctions mapped on the mm10 reference sequence were consistent with two previously reported junctions reported using the mm9 reference sequence [13]. [file 41021_2021_196_MOESM3_ESM.pdf]
