# Supplementary material for: Characteristic mutations induced in the small intestine of Msh2-knockout gpt delta mice
Source: Genes Environ. 2021 Jul 5;43:27. doi: 10.1186/s41021-021-00196-0 (PMC8256579; doi:10.1186/s41021-021-00196-0)
Supplement: Supplementary file 4 — Additional file 4: Figure S4. Positions of mutations in the gpt gene in the small intestine of Msh2-bearing mice (M(+/+)) carrying gpt and Msh2-KO mice (M(−/−)) carrying gpt. Potassium bromate (1.5 g/L) in the drinking water or the vehicle control (Control) was administered for 28 days. Numbers in the column next to the doses are animal identification numbers shown in Additional Table S1. Letters A, G, T, and C indicate base substitutions, and the number of letters in brackets are the number of clonal mutants. Arrows indicate the positions of insertion of base(s). d and (AA) indicates deletion of base and insertion of two adenine residues, respectively. Red letters in the gpt sequence indicate nucleotides whose mutations were induced. [file 41021_2021_196_MOESM4_ESM.pdf]

Fig. S4

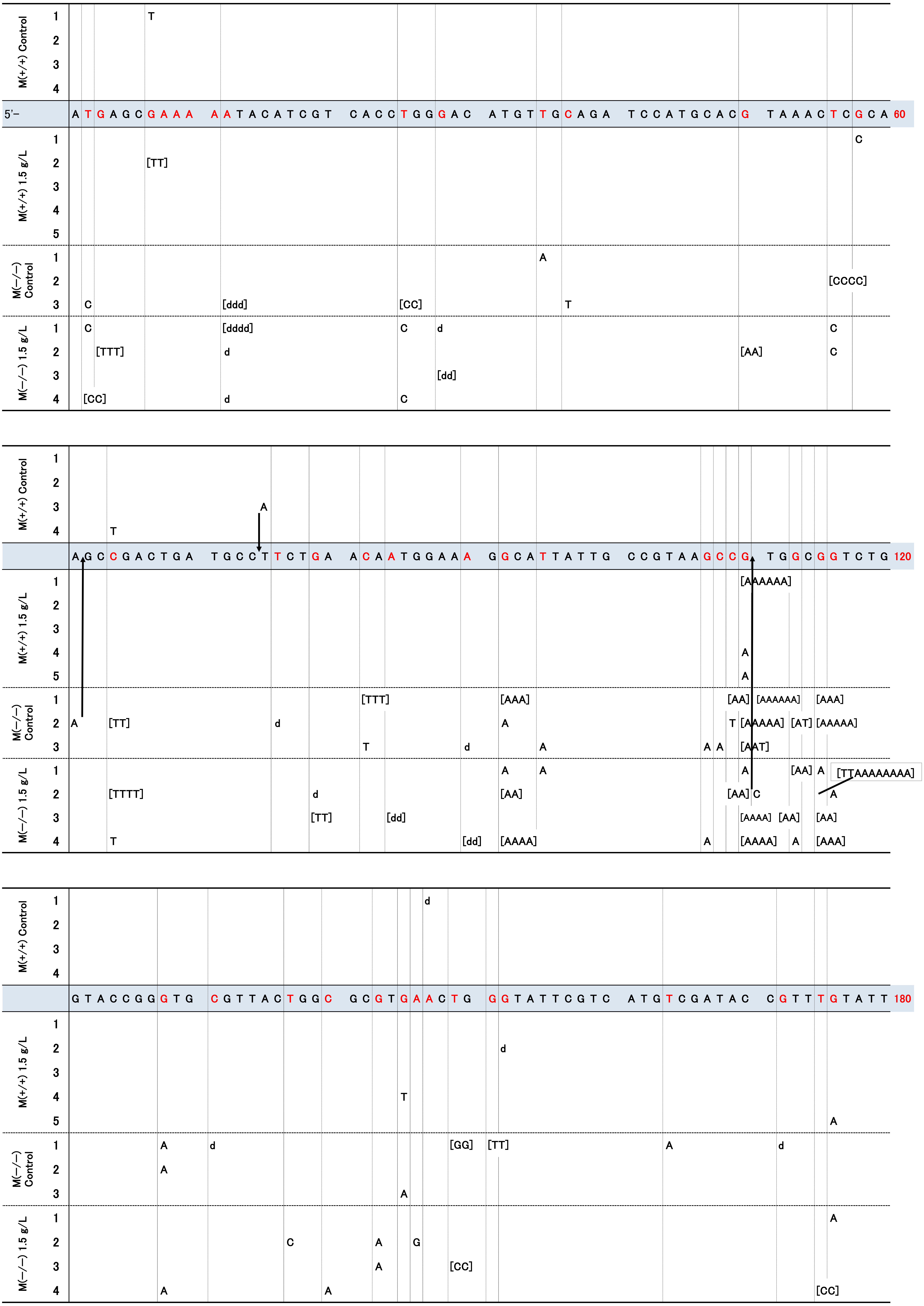
M(+/+) Control
5'–
A T G A G C G A A A A A T A C A T C G T C A C C T G G G A C A T G T T G C A G A T C C A T G C A C G T A A A C T C G C A 60
M(+/+) 1.5 g/L
M(−/−) Control
M(−/−) 1.5 g/L
T
C
[TT]
A
[CCCC]
C
[ddd]
[CC]
T
[dddd]
d
[TTT]
[AA]
[dd]
[CC]
A G C C G A C T G A T G C C T T C T G A A C A A T G G A A A G G C A T T A T T G C C G T A A G C C G T G G C G G T C T G 120
[AAAAAA]
[TTT]
[AAA]
[AA]
[AAAAAA]
[TT]
[AAAAA]
[AT]
[AT]
[AAT]
[TTAAAAAAAA]
[TTTT]
[TT]
[dd]
[AAAA]
G T A C C G G G T G C G T T A C T G G C G C G T G A A C T G G G T A T T C G T C A T G T C G A T A C C G T T T G T A T T 180
[GG]
[TT]
[CC]

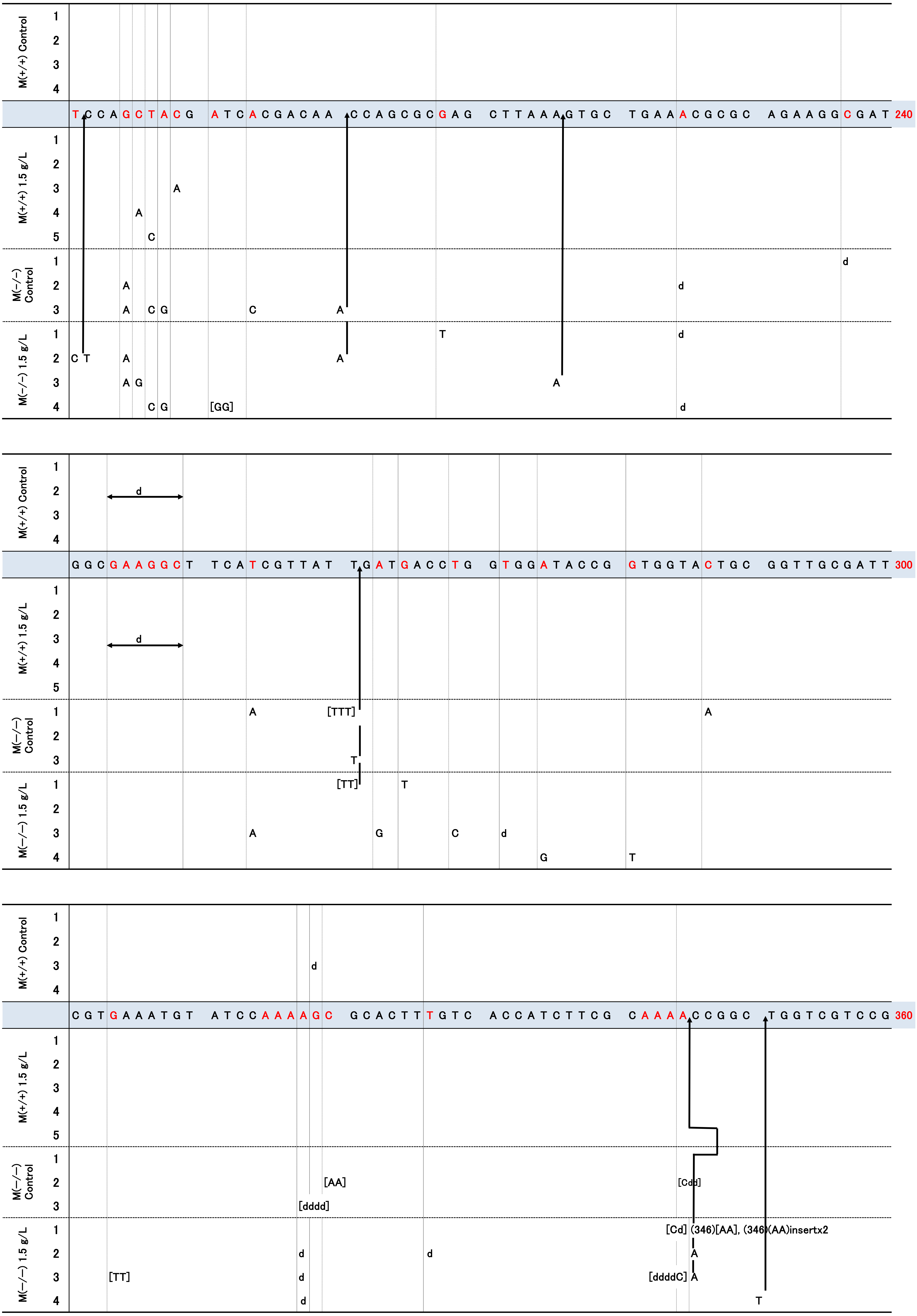
M(+/+) Control
M(+/+) 1.5 g/L
M(−/−) Control
M(−/−) 1.5 g/L
T C C A G C T A C G A T C A C G A C A A C C A G C G C G A G C T T A A A G T G C T G A A A C G C G C A G A A G G C G A T 240
G G C G A A G G C T T C A T C G T T A T T G A T G A C C T G G T G G A T A C C G G T G G T A C T G C G G T T G C G A T T 300
C G T G A A A T G T A T C C A A A A G C G C A C T T T G T C A C C A T C T T C G C A A A A C C G G C T G G T C G T C C G 360
[GG]
[TTT]
[TT]
[AA]
[dddd]
[Cdd]
[Cd] (346)[AA], (346)(AA)insertx2
[ddddC]

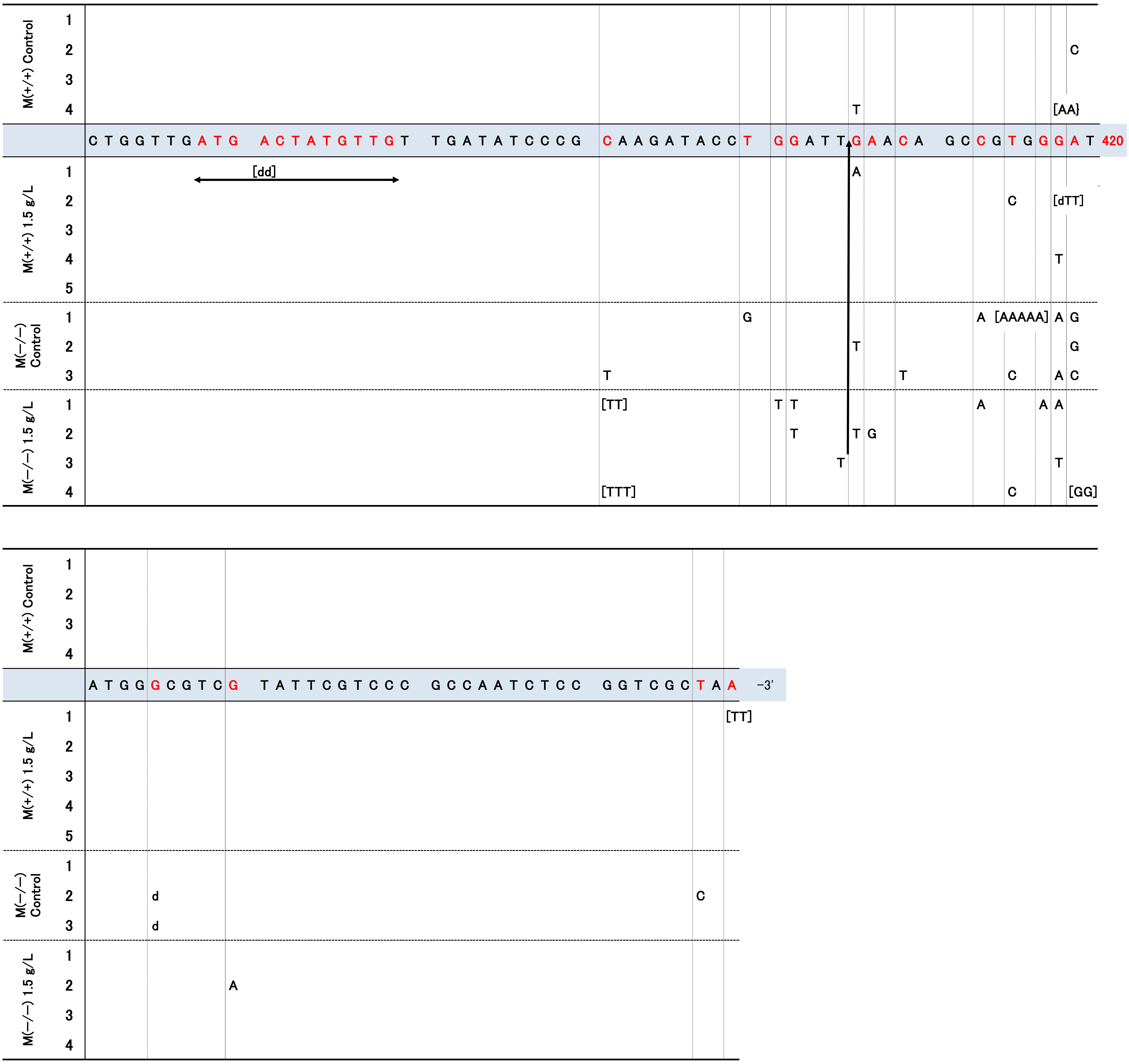
M(+/+) Control
M(+/+) 1.5 g/L
M(−/−) Control
M(−/−) 1.5 g/L
C T G G T T G A T G A C T A T G T T G T T G A T A T C C C G C A A G A T A C C T G G A T T G A A C A G C C G T G G G A T 420
[dd]
[AA}
[dTT]
[AAAAA]
[TT]
[TTT]
[GG]
A T G G G C G T C G T A T T C G T C C C G C C A A T C T C C G G T C G C T A A −3'
[TT]
