# Supplementary material for: Characteristic mutations induced in the small intestine of Msh2-knockout gpt delta mice
Source: Genes Environ. 2021 Jul 5;43:27. doi: 10.1186/s41021-021-00196-0 (PMC8256579; doi:10.1186/s41021-021-00196-0)
Supplement: Supplementary file 5 — Additional file 5: Table S1. Mutant frequencies in the small intestine of Msh2-bearing or -KO mice carrying gpt to which vehicle or potassium bromate was administered via the drinking water. [file 41021_2021_196_MOESM5_ESM.pdf]

Table S1. Mutant frequencies in the small intestine of *Msh2*-bearing or -KO mice carrying *gpt* to which vehicle or potassium bromate was administered via the drinking water.

Genotype	Dose	Animal ID and sex		Number of colonies Mutant	Total	Mutant frequency (10^{-5})	Average mutant frequency ± SD (10^{-5})	
Msh2+/+	0 g/L	1	♂	2	1,154,250	0.17	0.45	± 0.23
		2	♂	2	405,990	0.49		
		3	♀	2	494,235	0.40		
		4	♂	4	552,960	0.72		
		**Total**		**10**	**2,607,435**			
Msh2+/+	1.5 g/L	1	♂	12	911,325	1.32	0.83	± 0.58
		2	♂	7	1,187,325	0.59		
		3	♂	2	812,160	0.25		
		4	♀	4	256,230	1.56		
		5	♀	3	694,530	0.43		
		**Total**		**28**	**3,861,570**			
Msh2 −/−	0 g/L	1	♀	41	820,350	5.00	4.87 ** ##	± 1.33
		2	♂	34	975,375	3.49		
		3	♀	32	521,280	6.14		
		**Total**		**107**	**2,317,005**			
Msh2 −/−	1.5 g/L	1	♀	33	1,054,575	3.13	4.14 ** ##	± 1.43
		2	♂	42	1,145,850	3.67		
		3	♂	35	999,600	3.50		
		4	♂	39	624,060	6.25		
		**Total**		**149**	**3,824,085**			

** $P < 0.01$, significantly different compared with vehicle control *Msh2*-bearing mice (*Msh2+/+*, 0 g/L).

$P < 0.01$, significantly different compared with *Msh2*-bearing mice administered potassium bromate (*Msh2+/+*, 1.5 g/L).
